# Quality of Life in Workers and Stress: Gender Differences in Exposure to Psychosocial Risks and Perceived Well-Being

**DOI:** 10.1155/2017/7340781

**Published:** 2017-12-03

**Authors:** Simone De Sio, Fabrizio Cedrone, Donatella Sanità, Pasquale Ricci, Paola Corbosiero, Mario Di Traglia, Emilio Greco, Stephen Stansfeld

**Affiliations:** ^1^Research Unit of Occupational Medicine, Sapienza University of Rome, Rome, Italy; ^2^Course in Techniques of Prevention in the Workplace, Sapienza University of Rome, Rome, Italy; ^3^SAIMLAL Department, Sapienza University of Rome, Rome, Italy; ^4^Department of Public Health and Infectious Diseases, Sapienza University of Rome, Rome, Italy; ^5^Department of Neurology and Psychiatry, Sapienza University of Rome, Rome, Italy; ^6^Centre for Psychiatry, Wolfson Institute of Preventive Medicine, Barts and the London School of Medicine, Queen Mary University of London, London, UK

## Abstract

**Background:**

Quality of working life is the result of many factors inherent in the workplace environment, especially in terms of exposure to psychosocial risks.

**Objectives:**

The purpose of this study is to assess the quality of life with special attention to gender differences.

**Methods:**

The HSE-IT questionnaire and the WHO-5 Well-Being Index were administered to a group of workers (74 males and 33 females). The authors also used Cronbach's alpha test to assess the internal consistency of both questionnaires and the Mann–Whitney test to evaluate the significance of gender differences in both questionnaires.

**Results:**

The HSE-IT highlighted the existence of work-related stress in all the population with a critical perception regarding the domain “Relationships.” Furthermore, gender analysis highlighted the presence of two additional domains in the female population: “Demand” (*p* = 0,002) and “Support from Managers” (*p* = 0,287). The WHO-5 highlighted a well-being level below the standard cut-off point with a significant gender difference (*p* = 0.009) for males (18, SD = 6) as compared to females (14, SD = 6,4). Cronbach's alpha values indicated a high level of internal consistency for both of our scales.

**Conclusions:**

The risk assessment of quality of working life should take into due account the individual characteristics of workers, with special attention to gender.

## 1. Introduction

Psychosocial risks arise from the interaction between job content, work organization, technological and environmental conditions, and the employees' own competencies, resources, and needs [1]. All these elements can determine work-related stress. Furthermore, the new sociocultural and medical knowledge has raised growing awareness about the role of the work environment as one of the social determinants of health [2]. The consequences for the health of workers who are chronically subjected to the psychosocial risks and work-related stress phenomenon are manifold and they affect many organs and systems [3–12]. The European Agency for Safety and Health at Work (EU-OSHA) defines work-related stress as the feeling of unease and discomfort workers may experience when presented with work demands and pressures that are not matched to their knowledge and abilities and which challenge their ability to cope.

In Italy, Legislative Decree number 81 dated 9 April 2008 and following modifications and integrations introduced the obligation for Italian companies to assess work-related stress risk in view of protecting their workers' safety and health. A new risk has therefore been brought to the attention of the occupational physician: the risk of work-related stress; this type of risk has features that make it very difficult to manage because of the gap between scientific knowledge, preventive policies, and daily practices [13].

2010 saw the introduction of a set of guidelines, based on the Health & Safety Executive (HSE) Management Standards (MS) model, in order to meet the regulatory requirements. Based on this model, risk assessment is divided into two distinct phases: the first involving the analysis of objective data and the second assessing the subjective perception of workers through the Health and Safety Executive Indicator Tool (HSE-IT) questionnaire. It is worth pointing out that risk assessment of work-related stress should not be an end in itself but rather be conducted to plan appropriate corrective measures aimed at ensuring an effective management of psychosocial risks and creating a safe and healthy work environment so as to improve workers' welfare and the company's performance.

This research focuses on computer-using office workers who are exposed to psychosocial risks [14] derived from their job, as a result of repetitive data entry tasks [15], as well as from contact with the public which may represent an additional stress source [16–18]. The purpose of this research is to assess the quality of working life, especially in terms of exposure to psychosocial risks, in a group of workers who belong to the same company and share the same workplaces, with particular attention to gender differences that, according to EC regulations, deserve careful attention.

## 2. Methods

During the health surveillance activities carried out pursuant to the current legal framework in the years 2015/2016, the authors selected a sample from a population of *N* = 144 office workers, with an 8:30 a.m.–17:15 p.m. working time.

A clinical medical history questionnaire was administered to all subjects. The questionnaire was anonymous, with age and job seniority being the only details requested. The HSE-IT questionnaire and the WHO-5 questionnaire were also administered to all subjects.

The authors ensured a homogenous and comparable sample, according to confounding variables such as age and job seniority, using the statistical design of a stratified random sample (with gender as stratification variable). The sample size obtained included 74 male subjects and 33 female subjects (total *N* = 107).  Table 1 shows the structure of the sample.

The HSE-IT questionnaire was developed by the Health and Safety Executive [19, 20] and the Italian version was subsequently validated in our country [21]. The questionnaire is a useful tool designed to assess working conditions likely to cause work-related stress; it consists of 35 items rated on 5-point Likert scale, where higher scores indicate better working conditions and lower stress risk and define 7 different domains corresponding to as many primary factors of work-related stress risk.

*Demands*. They include such issues as workload, work patterns, and the working environment. 

*Control*. It focuses on workers' decision-making autonomy. 

*Support*. It is analysed in terms of “support from managers” and “support among colleagues” and includes the encouragement, sponsorship, and resources provided by the organization, line management, and colleagues. 

*Relationships*. They include promotion of positive working practices to avoid conflicts and deal with unacceptable behaviour. 

*Role*. It has to do with whether workers understand their role within the organization and whether the organization ensures that no conflicts occur. 

*Change*. It has to do with how organizational change (large or small) is managed and communicated in the organization.

The questionnaires were uploaded to the HSE Analysis Tool, specific software that analyses them and classifies the workers into four risk groups for each of the seven domains:

- Those below the 20th percentile (20% of the lowest reference values), for which corrective action is urgently required (red)
- Those below average (<50%) but still above the 20th percentile rank, for which corrective action is required (yellow)
- Those at or above average (≤50%) but below the 80th percentile and not requiring action (blue)
- Those at or above the 80th percentile, for which no corrective action is required (green)

Comparison with the benchmark was used to establish priorities for action and to set short- and long-term performance targets for each of the scales. The HSE Analysis Tool software was used to process collected questionnaires and three distinct profiles (total population, male population, and female population) were highlighted. The WHO-5 questionnaire was first presented by the WHO Regional Office in Europe at a 1998 WHO meeting in Stockholm as an element in the DEPCARE project on the measures of well-being in primary health care. Since then, the WHO-5 has been validated in a number of studies with regard to both clinical validity and psychometric validity [22]. The WHO-5 Well-Being Index is a questionnaire that measures current mental well-being (time frame: the previous two weeks). It is composed of 5 items rated on 6-point Likert scale, which indicate subjective quality of life based on positive mood (good spirits, relaxation), vitality (being active and waking up fresh and rested), and general interest (being interested in things).

Items explored by the questionnaire are listed as follows:

- I have felt cheerful and in good spirits.
- I have felt calm and relaxed.
- I have felt active and vigorous.
- I woke up feeling fresh and rested.
- My daily life has been filled with things that interest me.

The score is calculated by summing the score of the answer to each item. Possible responses to these prospects and the corresponding number of points were “all the time” (5), “most of the time” (4), “more than half the time” (3), “less than half the time” (2), “sometimes” (1), and “never” (0). A crude score below 13 indicates poor perceived well-being.

The authors also used Cronbach's alpha test, the Mann–Whitney test, a nonparametric statistical analysis for independent samples, and the Analysis of Covariance.

The first test is intended to assess the internal consistency and it is considered as a measure of scale reliability. The formula for the standardized Cronbach's alpha is

(1) $$\begin{array}{c} \alpha =\frac{N\cdot \overset{-}{c}}{\overset{-}{v}+\left(N-1\right)\cdot \overset{-}{c}}. \end{array}$$

The second test is intended to evaluate the significance of gender differences of both questionnaires. The statistical calculations were performed using SPSS software. The third test is intended to verify whether independent variables (age and gender) can significantly contribute to explaining the dependent variables, as assessed by the HSE-IT and the WHO-5 questionnaires.

All questionnaires were self-administered, collected, and checked to make sure they had been properly and fully completed. All subjects agreed with the processing of their personal data, stating their awareness of the presence of sensitive data, and they agreed to treat the data obtained by the protocol in an anonymous and collective way, through scientific procedures, according to the principles of the Declaration of Helsinki.

## 3. Results

### 3.1. HSE Questionnaire Evaluation

The results of HSE Analysis Tool showed the presence of work-related stress in office workers, highlighting a critical perception regarding the domain “Relationships” in all three samples, with the lowest average score obtained by women. Furthermore, gender analysis showed the presence of additional critical perceptions in the female population affecting the domains “Demand” and “Support from Managers” (Table 3).

The HSE-IT questionnaire obtained a Cronbach's alpha coefficient equal to 0,876 for the total sample, 0,885 for the male sample, and 0,830 for the female sample (summarized in Table 2), indicating a high level of internal consistency for all three samples; Mann–Whitney test demonstrated the statistical significance of gender differences affecting the domain “Demand” (*p* = 0,002). The HSE-IT risk profile of the total, female, and male samples and the relative statistical significance analysis of gender differences are summarized in Table 3.

### 3.2. WHO-5 Questionnaire Evaluation

The results of the WHO-5 questionnaire showed the presence of a well-being level slightly above the cut-off point in all three samples, with the lowest average score obtained by women, as summarized in Table 4.

The WHO-5 questionnaire obtained a Cronbach's alpha coefficient equal to 0,880 for the total sample, 0,880 for the male sample, and 0,860 for the female sample. All these values, summarized in Table 5, indicate a high level of internal consistency for both questionnaires in all three samples.

The Mann–Whitney test showed significant differences between genders (*p* = 0,009). The results of the WHO-5 questionnaire and the results of the statistical significance of gender differences are summarized in Table 4.

The Analysis of Covariance showed significant results for both questionnaires only for the independent variable “gender” (*p* = 0,002) and not for the covariate “age” (*p* = 0,44).

## 4. Conclusion

The analysis of the subjective perception of work-related stress risk and well-being produced interesting results:

1. The presence of the critical domain “Relationships” which affects both males and females
2. The presence of two critical domains, “Demand” and “Support from Managers,” only found in the female sample
3. The lowest well-being perception level for the female sample

This information is particularly useful to plan the corrective action best tailored to meet the company's needs.

Specifically, the transverse critical domain “Relationships” is formed by four distinct items, as reported in the following:

- Item 5: “I am subject to personal harassment in the form of unkind words or behaviour”
- Item 14: “there is friction or anger between colleagues”
- Item 21: “I am subject to bullying at work”
- Item 34: “relationships at work are strained”

In this domain, there are explicit questions that also refer to behaviours considered as unacceptable within the organization. These behaviours occur within the company and have a negative effect on workers' health [23], especially on women [24]. Indeed, the quality of interpersonal relationships in the workplace is one of the factors with the strongest potential to impact the health of workers [25]. These results underline the presence of reduced social support which may instead act as a protective factor for the worker, thus increasing the risk of vulnerability to other psychosocial risks [26].

Gender analysis showed that a corrective intervention for the domains “Demand” and “Support from Managers” is required for the female gender. The critical domain “Demand” reveals occurrences of excessive workload and includes issues such as having unachievable deadlines, having to work very fast, having to work very intensively, having to neglect some tasks because of other tasks to complete, being unable to take sufficient breaks, and having unrealistic time pressures. The domain “Support from Managers” refers to supportive feedback given to the workers, the possibility of relying on the line manager to help out with a work problem, talking to the line manager about something upsetting or annoying about work, the support through emotionally demanding work, and encouragement received by line managers at work.

The authors point out that, without gender analysis, the two additional critical domains highlighted in the female sample would have remained undetected as experienced by the authors themselves in a previous work [27].

Indeed, the study of the subjective perception of stress should include gender analysis; as a matter of fact, men and women use different coping strategies to manage stress [28, 29]. The physiological, biological, and cognitive differences between women and men are related to the social and psychological constructs which are inextricably connected. The social context in which gender differences in the workplace were investigated is relevant in this regard [30]. The division of labour in Italy has a more pronounced gender connotation than in other countries [31]. Family work remains an almost exclusive responsibility of women at all stages of life [32].

For these reasons, the authors agree on the idea that the highest vulnerability for the domain “Demand” could indicate the presence of “work-family conflict,” not investigated by the questionnaire used [33]. Female managers tend to report more quantitative demands than male managers [34], and more generally female workers across Europe report more demands than male workers [35] and this may reflect an interaction with demands from home shown by work-family conflict. Low support from managers may also mean that women are allowed less flexibility in dealing with demands and restructuring their work.

Work-family conflict [36] arises when participation in the work role and the family role are mutually incompatible in some respects. As a result, participation in one role is made more difficult by virtue of participation in the other role. The woman is a social element on whose shoulders weighs responsibility for parental and home care which may be in conflict with the demands that the working organization imposes on her.

To explain the presence of the critical domain “Support from Managers,” the authors refer to different scenarios. This support may have been requested and failed precisely in response to perceived problems regarding “Demands” and “Relationships.” Otherwise, this perception of the female population may be related to the presence of unacceptable behaviour, indicated by the presence of the critical domain “Relationships,” which could also indicate the occurrence of sexual harassments which women are more likely to suffer from.

In the risk assessment of quality of working life, the individual characteristics of the worker must be taken into account. One of the features that we should pay special attention to is the worker's gender.

## Figures and Tables

**Table 1:** Data on the total population and on the population by gender groups.

	Total sample *N* = 107	Male sample *N* = 74 (69,15%)	Female sample *N* = 33 (30,85%)
Mean age (SD)	52 (6,1)	52,28 (6,12)	52,56 (5,67)
Mean job seniority (SD)	14,46 (8,65)	15,14 (8,83)	13,3 (8,24)

**Table 2:** Cronbach's alpha coefficient of the HSE-IT for total, male, and female samples.

	Cronbach's alpha coefficient		
	Total sample	Male sample	Female sample
HSE-IT	0,876	0,885	0,830

**Table 3:** HSE profile of the 3 samples: mean values of the total sample, male sample, and female sample and statistical significance of gender differences by Mann–Whitney test.

HSE domain	Total sample Mean (SD)	Male sample Mean (SD)	Female sample Mean (SD)	*U*	*Z*	*p* value
Demand	3,22^b^ (0,61)	3,36^a^ (0,57)	**2,91** ^d^ (0,58)	749,500	3,17707	**p = 0,002**
Control	3,68^b^ (0,66)	3,70^b^ (0,69)	3,61^b^ (0,59)	1127,500	−0,62732	*p* = 0,564
Support from Managers	3,61^b^ (0,85)	3,69^a^ (0,78)	**3,45** ^c^ (0,98)	1045,000	−1,0887	*p* = 0,287
Support from Peers	3,94^a^ (0,73)	3,95^a^ (0,74)	3,93^a^ (0,70)	1164,000	−0,38111	*p* = 0,739
Relationships	**3,52** ^d^ (1,17)	**3,56** ^d^ (1,14)	**3,37** ^d^ (1,23)	1083,500	0,92412	*p* = 0,333
Role	4,36^a^ (0,51)	4,40^a^ (0,53)	4,29^b^ (0,46)	998,000	−1,50085	*p* = 0,145
Change	3,57^a^ (0,89)	3,68^a^ (0,85)	3,31^a^ (0,94)	920,000	−1,93775	*p* = 0,051

^a^Performance classified as very good (beyond the 80th percentile of the benchmark); ^b^performance classified as good, with potential for improvement; ^c^performance classified as requiring improvement; ^d^performance classified as requiring urgent improvement measures.

**Table 4:** WHO-5 questionnaire mean scores of total, male, and female samples and statistical significance of gender differences by Mann–Whitney test.

	Total sample Mean (SD)	Male sample Mean (SD)	Female sample Mean (SD)	*U*	*Z*	*p* value
WHO-5 score	16 (6)	18 (6)	14 (6,4)	772,500	−2,58688	**p = 0,009**

**Table 5:** Cronbach's alpha coefficient of the WHO-5 for total, male, and female samples.

	Cronbach's alpha coefficient		
	Total sample	Male sample	Female sample
WHO-5	0,880	0,880	0,860
